# Potential clinical biomarkers in rheumatoid arthritis with an omic approach

**DOI:** 10.1186/s13317-021-00152-6

**Published:** 2021-05-31

**Authors:** Yolima Puentes-Osorio, Pedro Amariles, Miguel Ángel Calleja, Vicente Merino, Juan Camilo Díaz-Coronado, Daniel Taborda

**Affiliations:** 1 Antioquia University, Medellín, Colombia; 2 University Hospital Virgin of Macarena, Sevilla, Spain; 3 Artmédica IPS, Medellín, Colombia; 4 Pharmacogenomic Center, Medellín, Colombia; 5 41009 Sevilla, Spain

**Keywords:** Rheumatoid arthritis, Genomics, Proteomics, Treatment, Omics, Biomarkers, Pharmacogenomics, Metabolomics, Polymorphism, Stages

## Abstract

**Objective:**

To aid in the selection of the most suitable therapeutic option in patients with diagnosis of rheumatoid arthritis according to the phase of disease, through the review of articles that identify omics biological markers.

**Methods:**

A systematic review in PubMed/Medline databases was performed. We searched articles from August 2014 to September 2019, in English and Spanish, filtered by title and full text; and using the terms "Biomarkers" AND “Rheumatoid arthritis".

**Results:**

This article supplies an exhaustive review from research of objective measurement, omics biomarkers and how disease activity appraise decrease unpredictability in treatment determinations, and finally, economic, and clinical outcomes of treatment options by biomarkers’ potential influence. A total of 122 articles were included. Only 92 met the established criteria for review purposes and 17 relevant references about the topic were included as well. Therefore, it was possible to identify 196 potential clinical biomarkers: 22 non-omics, 20 epigenomics, 33 genomics, 21 transcriptomics, 78 proteomics, 4 glycomics, 1 lipidomics and 17 metabolomics.

**Conclusion:**

A biomarker is a measurable indicator of some, biochemical, physiological, or morphological condition; evaluable at a molecular, biochemical, or cellular level. Biomarkers work as indicators of physiological or pathological processes, or as a result of a therapeutic management. In the last five years, new biomarkers have been identified, especially the omics, which are those that proceed from the investigation of genes (genomics), metabolites (metabolomics), and proteins (proteomics). These biomarkers contribute to the physician choosing the best therapeutic option in patients with rheumatoid arthritis.

## Relevance

- We could associate a better outcome in the patient’s treatments reducing unpredictability of the management.
- We made a review searching for the biomarkers associated with the different phases of rheumatoid arthritis.

## Introduction

Rheumatoid arthritis (RA) is a chronic autoimmune disease which is progressive and often disabling characterized by joint inflammation and pain; it requires monitoring of disease activity to decide the optimal treatment. Decreased quality of life, reduction of function and work participation are associated with mental and physical health of the patients. The use of biomarkers as monitors of disease development may aid providers improve non-biologic and biologic drugs [1]. Biomarkers have the capacity to enhance payment for medical and pharmacy policies related with the therapeutic management in immune system disorders and inflammatory diseases; they also has a potential impact on economic and clinical outcomes of treatment choices [2].

The aim of RA therapy is to keep and increase a prolonged state of health associated with quality of life by controlling symptoms, preventing structural joint damage, normalizing physical function, and getting better function in their activities. However, in patients with RA, treated either with methotrexate or biologic disease-modifying anti-rheumatic drugs (DMARDs), only 40–60% of them respond effectively as measured by at least 50% of improvement of signs and symptoms of the disease (by ACR criteria), whereas 15–30% develop adverse drug events [3].

On the other hand, in 2013 it was reported a systematic review of the International Journal of Rheumatology that approximately 50% of RA patients in Europe interrupted their biological therapy of the TNF inhibitor group during the first five years of use as a result of ineffectiveness or adverse drug reactions [4]. Similarly, a study in Boston at BRASS (Brigham and Women's Hospital Rheumatoid Arthritis Sequential Study) showed that 42% of patients with RA reported abandonment of their anti-TNF therapy due to ineffectiveness [5]. Hence, biomarkers could become a tool with greater capacity to predict the health results of therapeutic applications to expand them beyond early detection, timely evaluation of a prognosis, and selection of the most effective and safest therapy, as well as monitoring disease activity, resulting in greater preservation of joint space and motility of the RA patients [6].

RA is a major current public health problem, in terms of deterioration in the quality of life and the generation of high costs for the health system. Although in recent years, better health results have been achieved with the incorporation of disease modifying drugs (synthetic and biological), it is evident that there is a need to better redirect resources and take advantage of the window of opportunity of the disease.

Pharmacokinetics and pharmacodynamics of drugs, becomes a fundamental and additional instrument to those already existing, which allows us to determine the choice of a more effective and safer drug that meets the particular needs of the patient. Genetic information is expressed at the level of proteins and metabolites; therefore, it is necessary to determine specific predictors or biomarkers for different phases of rheumatoid arthritis, especially the OMICS that include pharmacogenomics, metabolomics, and proteomics (Fig. 2).

### Omics-based biomarkers classification

Biomarkers are interaction parameters that provide information on an objectively measurable physiological, biochemical, or morphological change that can be evaluable at the molecular, biochemical or cellular level and that acts as an indicator of a functional biological process or a pathogenic state, or as a response to medical treatment [7]. Biological markers are conceived as physiological signals induced by a xenobiotic, which is a cellular exposure, a precocious cellular response, or an inherent or acquired susceptibility [8].

Biomarkers are classified according to the information they provide and according to their nature. These biomarkers are important for identification of individuals in a population, that may be sensitive to a certain health problem. These kinds of biomarkers are classified as biomarkers of exposure, effect and susceptibility.

Exposure biomarkers evaluate the presence in an organism of an exogenous substance, a metabolite or product of the interaction between xenobiotic agent (natural or synthetic compounds of the environment that organism metabolizes and accumulates) and a molecule or target cell.

Prognostic Biomarkers report about progression of disease; this is, if disease improves or worsens after corresponding treatment. EphB4 membrane receptor is a prognostic biomarker of colon cancer [7].

Biomarkers of susceptibility are indicators of the inherited or acquired capacity of a given organism to respond to exposure to xenobiotic substances.

Biomarkers according to their nature are classified into omics that come from the study of genes (genomics), proteins (proteomics) and metabolites (metabolomics); epigenetics that come from changes that occur in DNA and that are related to some pathology, and microRNA molecules that are expressed in different amounts in either normal or cancerous cells (genomics/transcriptomics) [9].

According to the Food and Drug Administration, biomarkers are classified as follows [10, 11]:

- Diagnostic biomarker: used to detect or confirm presence of a disease or certain condition, or to identify individuals with a subtype of disease. For example: HbA1c is commonly the most used biomarker to diagnose prediabetes and diabetes [12].
- Prognostic biomarker: used to identify probability of a clinical event, disease recurrence or progression in patients with a diagnosis of a disease or medical condition of interest. For example: increasing prostate-specific antigen (PSA) as predictor of clinical progression for prostate cancer [13].
- Safety biomarker: used to indicate the likelihood, presence, or extent of toxicity as an adverse effect measured before or after exposure to a medical product or derived from environmental causes. For example: transaminases have been selected as biomarkers for potentially hepatotoxic drugs [14].
- Monitoring biomarker: measured in series to assess the level of a disease or medical condition, or the evidence of exposure to (or the effect of) a medical product or environmental agent. For example: B-type natriuretic peptide as a measure of vascular and ventricular function in pediatric pulmonary arterial hypertension [15].
- Pharmacodynamic response biomarker: used to demonstrate that in exposition to medical products or environmental causes in an individual there is a biological reaction. For example: International Standardized Ratio (INR) for anticoagulant treatment, which has special interest in the adjustment of drug [16].

Ideal biomarkers should provide diagnostic, prognostic, and therapeutic information; additionally, they have to be obtainable from patient's clinical data, and should possess chemical-analytical characteristics such as:

- High specificity: measurement of a biomarker must be specific to a disease.
- Specimen: collection of samples should be minimally invasive. For example, saliva is better than urine and urine better than blood.
- Representativeness: levels of biomarkers in the selection sample should be representative of levels of biomarkers in the organism.
- Stability: kinetics must be known [17].

Regarding pharmacological safety of patients, ideal biomarkers should be aimed at health care processors. So, it is advisable to guide pharmacotherapeutic follow-up and programs for appropriate use of drugs through these pointing elements (tracers or markers). In this sense, detection of these elements is highly recommended, by means of information systems (systematized monitoring of warning signals), such as: identification of some medications, laboratory tests, symptoms or diagnoses and medical notes or phrases in clinical histories, known as markers or bookmarks [18].

## Materials and methods

**Fig. 1:**
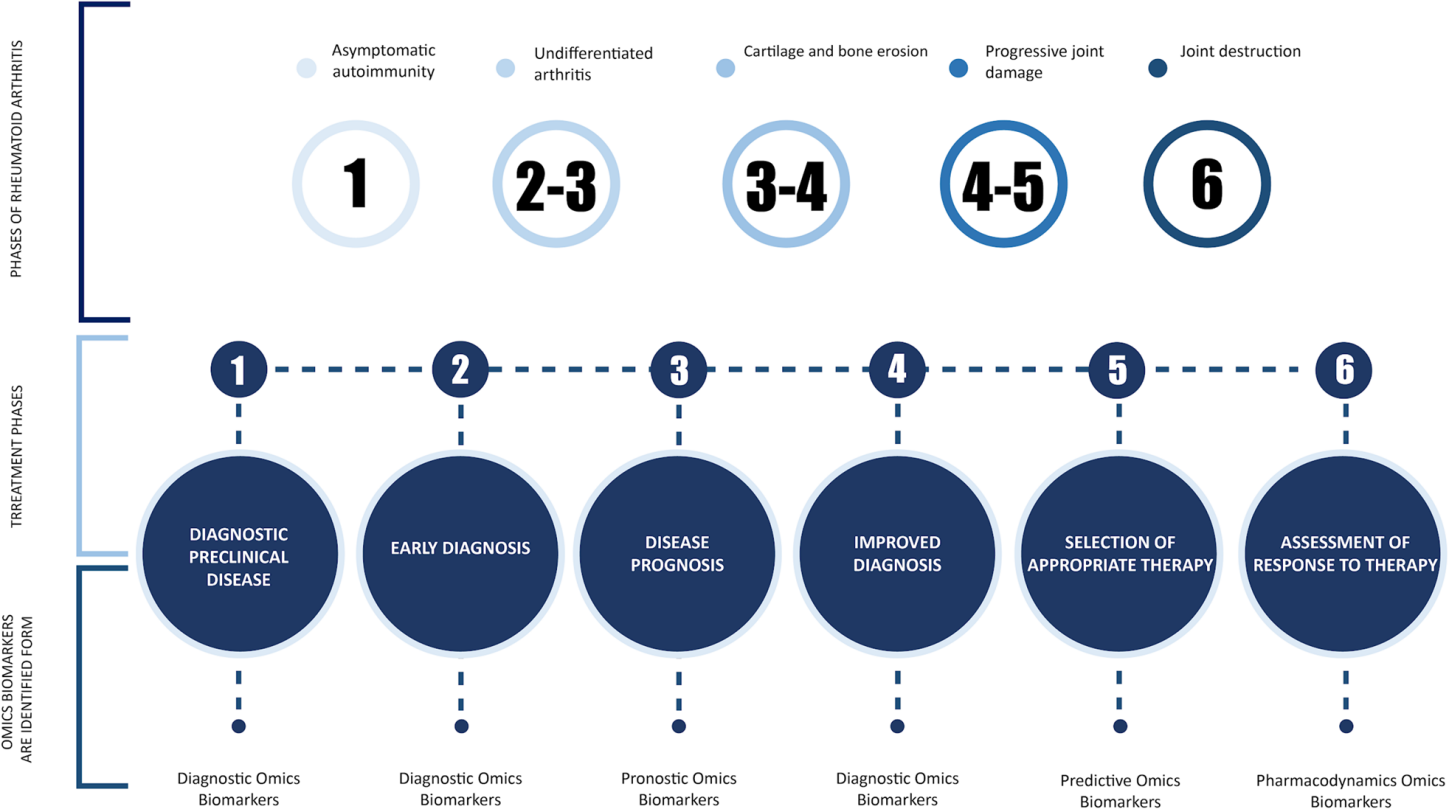
Phases of rheumatoid arthritis, treatment phases and omics biomarkers

A systematic review was performed in PubMed/Medline databases. We searched articles from August 2014 to September 2019, in English and Spanish, filtered by title, full text, and using the terms "biomarkers" AND "Rheumatoid Arthritis". Inclusion criteria defined articles that reported biomarkers in different phases of rheumatoid arthritis and drug specific uses (Fig. 1).

Data extraction was performed on articles that met the inclusion criteria. Articles were downloaded and analyzed according to predefined eligibility criteria in a systematic review database. A format was created with reference, omics biomarker, phase of the disease, and a short description of potential use in clinical practice.

## Results

A total of 122 articles were included, only 92 met established criteria for review purposes and 17 relevant references about the topic were included. Therefore, it was possible to identify 196 potential clinical biomarkers: 22 non-omics, 20 epigenomics, 33 genomics, 21 transcriptomics, 78 proteomics, 4 glycomics, 1 lipidomics and 17 metabolomics. Figure 1 shows screening carried out concluding in the identification of different types of omics biomarkers.

**Fig. 2:**
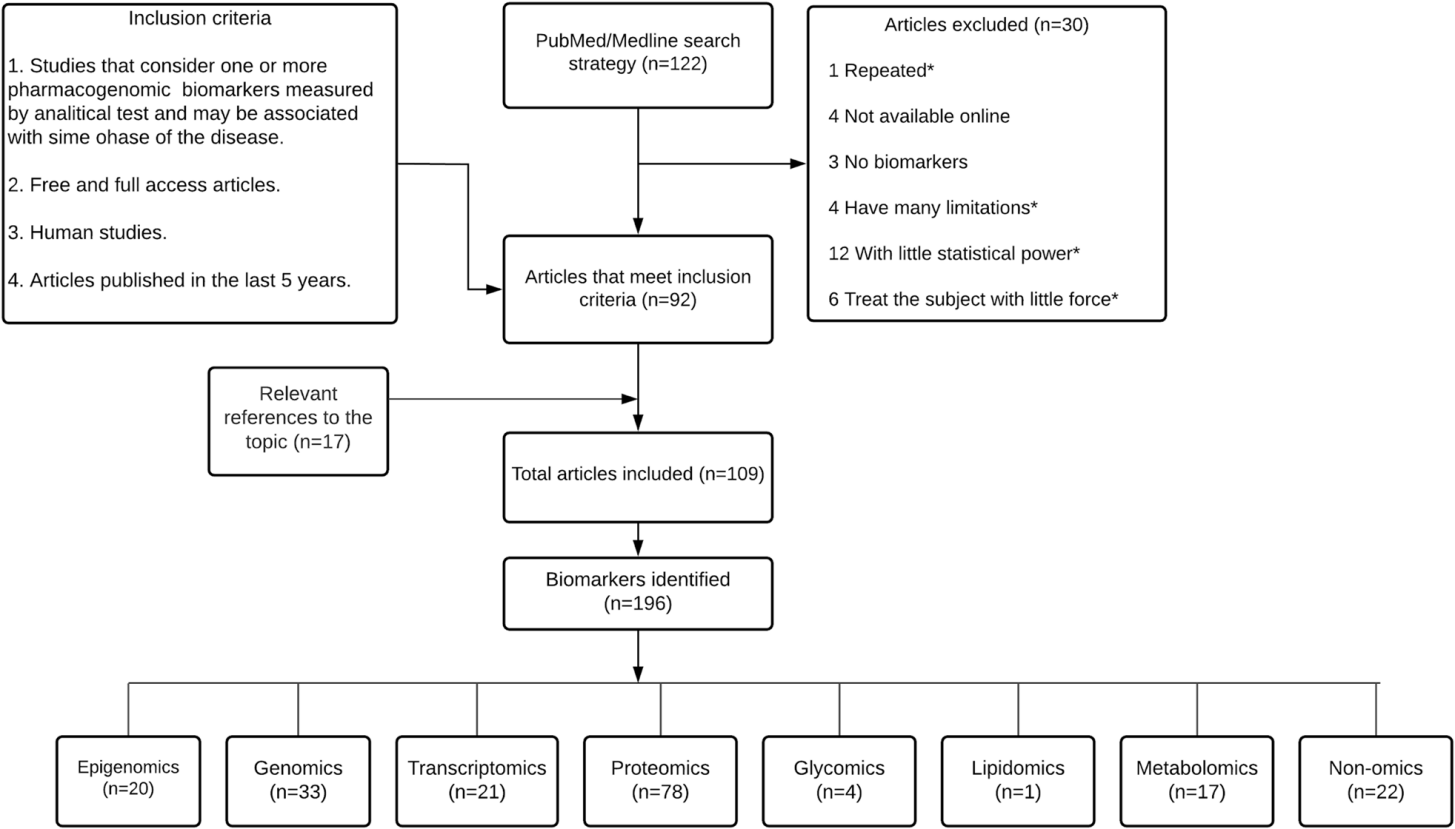
Flow diagram for screening and evidence selection. *The repeated article was excluded due to the biomarker having a different end up, and it may disturb the results of the study. *The articles that had little statistical power were not conclusive, and the statistical evaluation did not confirm the use of the biomarker. *The articles that had many limitations were excluded because the impossibility to find the optimal selection method, and factor omission which can affect the outcome. *The articles related to treating the subject with little force were also excluded due to lack of information between the treatment and the use of the biomarkers.

In addition, Fig. 2 shows different states of rheumatoid arthritis from the beginning with asymptomatic autoimmunity until joint destruction. Also, six phases of RA treatment are shown, and types of omics biomarkers are identified.

Consequently, identifying and monitoring biomarkers in different phases of disease will help to improve diagnosis, treatment, and quality of life of patients with RA.

Thus, this review identified both omic and non-omic biomarkers in each phase of RA. Table 1 shows a brief description of each biomarker and number of biomarkers identified in each phase of rheumatoid arthritis. For example, in phase 1 of RA were identified twenty-three biomarkers, in phase 2 seventeen, in phase 3 forty-one, in phase 4 eighteen, in phase 5 forty-nine, and in phase 6 forty-one.

**Table 1:** Identification of potential biomarkers for rheumatoid arthritis with omic-approaches

	Biomarker	Kind	Description	References
PHASE 1	HLA-DRB1*04 and *03 allelic groups	Genomics	Association with genetic susceptibility to RA in a female population in Bosnia and Herzegovina	[23, 123]
PHASE 1	DRB1*01/DRB1*15 and DRB1*07/DRB1*16 genotypes	Genomics	Protective factor for RA in a female population in Bosnia and Herzegovina	[23]
PHASE 1	HLA-DRB5 gene variants	Genomics	Protective factor for RA in a female population in Bosnia and Herzegovina	[23]
PHASE 1	TNFSF10 gen	Genomics	Protective role in eRA, however, it has the effect to promote the disease	[38]
PHASE 1	IL-12A rs2243115 GG genotype	Genomics	Significant association with increased risk of RA (RF negative patients)	[100]
PHASE 1	IL-12B rs3212227 AC and AC + CC genotypes	Genomics	Associated with RA risk in older patients, RF positive patients and ACPA negative patients	[100]
PHASE 1	IL-10 rs1800872 A/C polymorphisms	Genomics	Association with risk of RA in East Chinese Han patients	[118]
PHASE 1	Members of the S100 protein family of calcium-binding proteins, S100A8 (Calgranulin A), S100A9 (Calgranulin B) and S100A12 (Calgranulin C)	Proteomics	Discrimination between RA and other inflammatory arthritides. The calgranulin C is the unique protein of this family that discriminate significatively RA and PsA	[24]
PHASE 1	Collagen type II (CII), collagen type IX (CIX) and collagen type XI (CXI)	Proteomics	Serum levels of CII, CIX and CXI antibodies can serve as clinical diagnostic indicators. Patients with antibodies Cll are correlated with a phenotype of increased inflammation and early joint destruction	[35, 57]
PHASE 1	Interleukin 1 (IL-1)	Proteomics	Clinic risk factor predisposing to RA	[96, 101]
PHASE 1	30 metabolites	Metabolomics	Discrimination between RA patients and healthy subjects	[31]
PHASE 1	COL14A1 and CXCL12 genes	Transcriptomics	Overexpression in RA patients	[38]
PHASE 1	C-reactive protein (CRP)	Non-omics	Biomarker of elevated systemic inflammation in patients with RA. High serum value is a prognosis that indicates progressive bone erosion	[37, 55, 68, 70, 71, 75, 77, 79, 109, 116]
PHASE 1	Rheumatoid factor (RF)	Non-omics	Present (IgM isotype) in approximately 70–80% of patients with confirmed RA, with a sensitivity of 65–80% and 85% specificity for diagnosis. Considered useful in early stages of the disease to predict the development of erosions and the presence of the IgA isotype is associated with extra-articular manifestations. Aggressive development of the disease and decreased response to anti-TNF therapy have been reported at high levels	[42, 53, 57, 68, 74, 75, 99, 109, 121, 124]
PHASE 1	Anti-citrullinated protein antibodies (ACPA)	Non-omics	Biomarker more sensitive (60–80%) and specific (95–98%) for the diagnosis of RA than RF. ACPA + patients develop a more aggressive and erosive progressive disease clinical phase compared to ACPA- patients; Positivity has been associated with a better response to treatment in early stages but medication-free remission is less frequent. Baseline levels can be predictive for the response to methotrexate and ACPA + subjects are associated with a better response to abatacept independent of disease activity. It has been detected in healthy patients therefore increases the risk of developing RA by 5% in the next 5 years	[42, 51, 53–57, 65, 68, 74, 75, 99, 109, 110, 112, 121]
PHASE 1	Anticarbamylated protein (anti-CarP) antibodies	Non-omics	Association with rapid radiological damage and severe course of the disease independent of the ACPA value. In patients ACPA—it is associated with the development of arthralgias	[57, 65, 74, 109, 112]
PHASE 1	Regulatory B lymphocytes (Breg)	Non-omics	Protective role in RF + patients (Lower T2/Breg levels)	[62]
PHASE 1	Erythrocyte Sedimentation Rate (ESR)	Non-omics	Nonspecific indicator of the amount of inflammation in the elevated body in patients with RA. It correlates with CRP with radiographic progression and these indices have been incorporated into the composite scores that are generally used to predict damage	[68, 70, 75, 77, 79, 109]
PHASE 2	MicroRNAs (miRNAs): miR 361-5p	Epigenomics	Elevated levels in the serum of patients with early stage of the disease	[20]
PHASE 2	Human Ficolin-2 protein	Proteomics	Increased levels in RA patients	[125]
PHASE 2	Matrix metalloproteinase-1 (MMP-1)	Proteomics	Elevated baseline MMP-1 levels are significantly correlated with radiographic progression	[38, 109]
PHASE 2	Interleukin-7 receptor subunit alpha (IL7R)	Proteomics	Possible applications in the diagnosis and therapy of RA	[28]
PHASE 2	C–C motif chemokine 5 (CCL5)	Proteomics	Prediction of a negative impact in the development of RA	[28]
PHASE 2	Resistin	Proteomics	High level in RA patients	[98]
PHASE 2	Malondialdehyde (MDA)	Metabolomics	Increased in patients with RA	[70, 112]
PHASE 2	3-hydroxyisobutyrate, acetate, NAC, acetoacetate, and acetone levels	Metabolomics	Discrimination between RA and healthy subjects	[87]
PHASE 2	Valine, isoleucine, lactate, alanine, creatinine, GPC APC and histidine levels	Metabolomics	Decreased in RA patients	[88]
PHASE 2	Arginine, aspartic acid, glutamic acid, serine, phenylalanine, threonine, lysine	Metabolomics	Higher plasma concentration of arginine, aspartic acid, glutamic acid, serine, phenylalanine, threonine in RA patients than control was demonstrated while concentration of lysine was lower in RA patients	[95]
PHASE 2	Malondialdehyde-Acetaldehyde (MAA)	Metabolomics	Increased in RA patients. Furthermore, is associated to cardiovascular risk	[112]
PHASE 2	Glycan GP1	Glycomics	Putative diagnostic biomarker for RA in the Han Chinese population	[78]
PHASE 2	21 N-glycans	Glycomics	Discrimination between RA patients and HS	[51]
PHASE 2	Sulfated IgG N-glycans (SGm1 y SGm2)	Glycomics	Discrimination between RA and HS (sensitivity of 84% and specificity of 86%). Biomarkers for the classification of both RF negative and ACPA negative. (precision 93% y 95% in RA patients)	[52]
PHASE 2	Anti-mutated citrullinated vimentin (anti-MCV)	Non-omics	Significant correlation with ACPA (r = 0.73). comparable value to ACPA for RA early diagnosis, with lower sensitivity and specificity	[54, 99]
PHASE 3	MicroRNAs (miRNAs): miR 223‐3p and miR 16‐5p	Epigenomics	Prediction of disease outcome in eAR	[43]
PHASE 3	miR-642b-5p, miR-483-3p, miR-371b-5p (up-regulated) and miR-25-3p, miR-378d (down-regulated)	Epigenomics	Association with devolopment RA in undifferentiated arthritis patients after 4 years	[46]
PHASE 3	Hypo-methylation in 4 genes (FCRLA, CCDC88C, BCL11B and APOL6)	Epigenomics	Association with RA progression	[50]
PHASE 3	M1V variant SNP (rs3764880, A>G)	Genomics	Association with good activity of disease and classifies patients that require less therapeutic interventions	[58]
PHASE 3	Genetic variant (rs7607479) of the SPAG16 gene	Genomics	Protective role for radiological progression	[91]
PHASE 3	BF*S07 allotype of complement factor B	Genomics	Significantly associated with extra-articular manifestations (EAM) in brazilian RA patients	[99]
PHASE 3	B cell antigen receptor complexassociated protein alpha chain (CD79A)	Proteomics	Correlation with joint destruction	[28]
PHASE 3 PHASE 6	Interleukin 6 (IL-6)	Proteomics	Association with joint erosive progression. Patients with high levels might need an intensive treatment	[1, 36, 47, 51, 79, 98, 101, 116]
PHASE 3	Casein kinase 2 interacting protein 1 (CKIP-1) and a micro RNAs 214	Proteomics and transcriptomics	Biomarkers that Predict the progression of bone erosion	[37]
PHASE 3	C-telopeptide of type I collagen (CTX-I)	Proteomics	High values ​​reflect association with RA active and rapid joint destruction	[40, 69, 109]
PHASE 3	C-telopeptide of type II collagen (CTX-II)	Proteomics	High level of CTX II are associated with greater progression of joint damage in patients with RA	[40, 109]
PHASE 3	Receptor Activator for Nuclear Factor κ B Ligand (RANKL)	Proteomics	Prediction of radiological progression in eRA patients	[55, 69]
PHASE 3	Interleukin-13 (IL-13)	Proteomics	Along with IL-17, it could be of better use than RF and ACPA for predicting the state of eAR activity	[56]
PHASE 3	CD4 + T-cell-derived CD161 + CD39 + and CD39 + CD73 + microparticles	Proteomics	Association with disease progression (high levels)	[64]
PHASE 3	Osteoprotegerin (OPG)	Proteomics	Prediction (RANKL/OPG ratio) of joint damage in 5 and 11 years in patients without early treatment	[69]
PHASE 3	C-X-C motif chemokine 13 (CXCL13)	Proteomics	High baseline CXCL13 levels are associated with a higher probability of remission after 2 years. Further, high concentrations in plasma indicate that patient can respond better to an early more aggressive treatment	[73]
PHASE 3	Cartilage oligomeric matrix protein (COMP)	Proteomics	Association with degradation of articular cartilage	[74]
PHASE 3	Heme oxygenase-1 (HO-1)	Proteomics	Biomarker for bone metabolism in patients with RA and ankylosing spondylitis	[77]
PHASE 3	Bone morphogenetic protein (BMP)	Proteomics	Biomarkers for bone metabolism in patients with RA and ankylosing spondylitis	[77]
PHASE 3	Orosomucoid (ORM)1, ORM2 and soluble CD14 (sCD14)	Proteomics	Association with disease activity, furthermore ORM2 predicts the radiological progression	[93]
PHASE 3	Adiponectin, Visfactin	Proteomics	Correlation with increased radiological progression	[98, 109]
PHASE 3	Vascular endothelial growth factor (VEGF)	Proteomics	High levels are significantly correlated with radiological progression after 1 year	[109]
PHASE 3	Angiopoietin-1	Proteomics	Prediction of joint damage after 1 year	[109]
PHASE 3	Cartilage oligomeric matrix protein (COMP)	Proteomics	Predictions of joint damage at 1,2 and 5 years	[109]
PHASE 3	Human serum amyloid A (SAA)	Proteomics	Correlation with radiological progression. This protein reflects systemic and local inflammation	[109]
PHASE 3	Leptin	Proteomics	Association with decreased radiological progression	[109]
PHASE 3	C–C motif chemokine 11 (CCL11)	Proteomics	Association with decreased radiological progression	[109]
PHASE 3	Anti-peptidyl-arginine deaminase 3 (PAD3)	Proteomics	Association with severe radiological damage	[112]
PHASE 3	Coronary artery calcium	Metabolomics	Association with cardiovascular risk assessment in RA patients	[21]
PHASE 3	Vitamin K homologs: MK-4, MK-7 y PK	Metabolomics	Correlation with disease activity (lower levels in RA patients)	[68]
PHASE 3	Cholesterol, lactate, acetylated glycoprotein, and lipid signatures	Metabolomics	Prediction of disease severity	[85]
PHASE 3	Pigment epithelium-derived factor (PEDF)	Transcriptomics	Association with obesity in RA patients that influences the goal of remission	[41]
PHASE 3	313 differentially expressed genes (232 up-regulated genes and 81 down-regulated genes)	Transcriptomics	Association between inflammatory and immune with RA progression	[50]
PHASE 3	Model of FKBP1A, FGF12, ANO1, LRRC31, and AKR1D1	Transcritomics	The model is useful for efficiently predict the response to infliximab therapy in RA	[59]
PHASE 3	Signal transducer and activator of transcription 3 (STAT3)	Transcriptomics	Prediction of RA progression in ACPA negatives patients	[83]
PHASE 3	Platelet/lymphocyte ratio (PLR)	Non-omics	Discrimination between RA patients and rheumatoid arthritis-associated interstitial lung disease (RA-ILD) patients and for distinguishing healthy subjects	[22]
PHASE 4	Circular RNAs hsa_circ_0044235	Epigenomics	Discrimination between RA and systemic lupus erythematosus (SLE)	[39]
PHASE 4	Serum amyloid A4 and vitamin D binding protein	Proteomics	Selection of patients with rheumatoid arthritis from healthy controls	[19]
PHASE 4	14–3-3η proteins	Proteomics	Increased levels in RA patients. Further it is associated with joint damage. Determination of this protein with RF y ACPA increases the diagnostic rate (72%)	[51, 74, 112, 121]
PHASE 4	Binding immunoglobulin protein (BiP)	Proteomics	Discrimination between RA patients and healthy subjects	[57]
PHASE 4	Presepsin and procalcitonin	Proteomics	Identification of infections in patients with RA (presepsin has better infectious reflective status than procalcitonin)	[79]
PHASE 4	MMP7, PARC y SP-D biomarker signature	Proteomics	Association with Interstitial lung disease in RA	[114]
PHASE 4	γ-inducible protein 10 (IP-10)/CXCL10	Proteomics	Association with Interstitial lung disease in RA	[122]
PHASE 4	Matrix metalloproteinase-7 (MMP-7)	Proteomics	Discrimination between RA patients and rheumatoid arthritis-associated interstitial lung disease (RA-ILD)	[122]
PHASE 4	Histidine, methionine, asparagine and threonine	Metabolomics	Discrimination between RA and psoriatic arthritis	[86]
PHASE 4	Signal transducer and activator of transcription 1 (STAT1) signature	Transcriptomics	High levels in RA patients. Useful for Discrimination between RA patients and Osteoarthritis patients	[34]
PHASE 4	Mitogen-activated protein kinase kinase kinase 3 (MAP3K3) gene	Transcriptomics	Discrimination between RA and PsA	[82]
PHASE 4	CD117 + and CD138 + cells	Non-omics	Discrimination between psoriatic arthritis (PsA) patients and RA patients in the context of ACPA negativity	[27]
PHASE 4	Natural Killer (NK) cell (CD3 + CD56 +)	Non-omics	Discrimination between RA patients and chronic chikungunya arthritis patients	[60]
PHASE 4	Perforin + NK cells	Non-omics	Discrimination between RA patients and chronic chikungunya arthritis patients	[60]
PHASE 4	Diagnostic algorithm combining plasma/serum ACPA and hydroxyproline	Non-omics	Discrimination specific and sensivity between early stage osteoarthritis, early rheumatoid arthritis, other non-RA inflammatory joint diseases and good skeletal health and detection	[115]
PHASE 5	MicroRNAs (miRNAs): miR-132, miR-146a y miR-155	Epigenomics	Low baseline levels can be used to predict the positive response to MTX after 4 months of therapy	[33]
PHASE 5	MicroRNAs (miRNAs): miR-23 y miR-223	Epigenomics	Association with negative response to combined anti-TNFα/ DMARDs therapy and as biomarkers of response to combined anti-TNFα/DMARDs therapy (so that their levels are indicative of the efficacy of the treatment and also of the degree of response)	[113]
PHASE 5	SNP NUBPL (rs2378945)	Genomics	Significant association with a poor response to etanercept in patients with Spanish and Greek ancestry	[29]
PHASE 5	SNP CD84 (rs6427528)	Genomics	Possibly associated with the response to etanercept in patients with Spanish and Greek ancestry	[29]
PHASE 5	C3435T (rs1045642) SNP in ABCB1	Genomics	Association with the risk of poor response to methotrexate	[66]
PHASE 5	Two SNP (rs6028945) and (rs7305646)	Genomics	Prediction of response to anti-TNF therapy	[73]
PHASE 5	SNP (rs6427528) of the CD84 gene	Genomics	Association with good response to etanercept	[92]
PHASE 5	HLA-DRB1* haplotypes 04–04, 04–01 and 04–11	Genomics	Significantly associated with usage of T Cell Receptor Beta Variable 25–1 (TRBV25), higher disease activity at the onset of disease and poor response to DMARD	[94]
PHASE 5	SNP (rs6427528) in CD84 gene	Genomics	Association with positive response to etanercept, but not adalimumab and infliximab in patients of European descent	[103]
PHASE 5	SNP (rs3794271) in PDE3A-SLCO1C1 locus	Genomics	Association with positive response to infliximab and etanercept, but not adalimumab in Spanish and Danish patients	[104]
PHASE 5	SNP (rs113878252) in MED15 gene	Genomics	Association with negative response to etanercept in European Caucasian patients with grandparents born in Spain	[105]
PHASE 5	SNP (rs6941263) in the ARMC2 locus	Genomics	Association with global negative response to anti-TNF therapy in European Caucasian patients with grandparents born in Spain	[105]
PHASE 5	SNP (rs6065221) in the MAFB locus	Genomics	Association with negative response to etanercept and infliximab in European Caucasian patients with grandparents born in Spain	[105]
PHASE 5	SNP (rs10919563) in the PTPRC locus	Genomics	Association with positive response to etanercept, adalimumab and infliximab in patients with European ancestry especially among those seropositive for ACPA or RF	[106]
PHASE 5	SNP (rs1800896) in the IL10	Genomics	Association with response to etanercept, adalimumab and infliximab at 3 months	[107]
PHASE 5	SNP (rs 6,683,595) in the PTPRC	Genomics	Association with positive response to etanercept, adalimumab and infliximab at 6 months in patients of Spanish Caucasian or Greek Caucasian descent	[107]
PHASE 5	SNP (rs11591741) in the CHUK	Genomics	Association with positive response to adalimumab and infliximab at 3 months in patients of Spanish Caucasian or Greek Caucasian descent	[107]
PHASE 5	Single-nucleotide polymorphism (SNP) TNF-α − 308 G > A (rs1800629)	Genomic	Association with a poor response to infliximab, etanercept and adalimumab. However, patients who carry the G allele respond positively to biological therapy	[53, 124]
PHASE 5	9-protein signature	Proteomics	Association with a decreased chance (6/9) achieving sustained drug-free remission after initiation of tocilizumab plus methotrexate therapy in DMARD-naive patients with early RA	[44]
PHASE 5	14-protein signature	Proteomics	Association with a decreased chance (6/14) achieving sustained drug-free remission after initiation of tocilizumab plus methotrexate therapy in DMARD-naive patients with early RA	[44]
PHASE 5	13-protein signature	Proteomics	Association with a decreased chance (5/13) achieving sustained drug-free remission after initiation of tocilizumab plus methotrexate therapy in DMARD-naive patients with early RA	[44]
PHASE 5	Osteopontin	Proteomics	Serum levels before treatment predict the clinical remission for tocilizumab therapy but not for clinical remission induced for infliximab therapy	[51]
PHASE 5	TTTT B lymphocyte stimulator promoter haplotype (TTTT BLyS)	Proteomics	Significant association with good response to rituximab for seropositive RA patients after anti-TNF agents have failed	[53]
PHASE 5	Favorable Fcγ receptor III (FcγRIII) genotype	Proteomics	Prediction of positive response to treatment with Rituximab	[53]
PHASE 5	Cluster of differentiation 20 (CD20)	Proteomics	Prediction of response to rituximab therapy (significantly high values predict a negative response)	[1, 53, 112]
PHASE 5	15-protein signature	Proteomics	Association with response to IFX	[81]
PHASE 5	8-protein signature	Proteomics	Association with response to ADA	[81]
PHASE 5	8-protein signature	Proteomics	Association with response to IFX + ADA	[81]
PHASE 5	C-X-C motif chemokine 10 (CXCL10) and C-X-C motif chemokine 13 (CXCL13)	Proteomics	Baseline CXCL10 and CXCL13 levels are associated with favorable response to anti-TNF therapy (adalimumab or etanercept) at 14 weeks	[120]
PHASE 5	Erythrocyte folate levels	Metabolomics	Association with a poor response to MTX (Lower baseline levels)	[66]
PHASE 5	Histamine, glutamine, xanthurenic acid, and ethanolamine	Metabolomics	Association with anti-TNF therapy as responders and non-responders with infliximab and etanercept	[89]
PHASE 5	Increased levels of isoleucine, leucine, valine alanine, glutamine, tyrosine, and glucose, and decreased levels of 3-hydroxybutyrate	Metabolomics	Expressed in patients with good response before treatment with etanercept	[90]
PHASE 5	Type I interferons (IFNs) signature	Transcriptomics	Discrimination between responders and non-responders patients to MTX treatment for the first time after 6 months. High titles are also associated with a poor response of Infliximab at 12 y 22 weeks. Similarly, classifies non-responders patients to treatment of rituximab. In preclinic phases represents an independent risk clinical factor for predicter RA	[32, 55, 112, 117]
PHASE 5	9 (8 up-regulated, 1 down-regulated) signature genes	Transcriptomics	Prediction of sustained drug-free remission after initiation of tocilizumab plus methotrexate therapy in DMARD-naive patients with early RA	[45]
PHASE 5	7 (6 up-regulated, 1 down-regulated) siganture genes	Transcriptomics	Prediction of sustained drug-free remission after initiation of tocilizumab in DMARD-naive patients with early RA	[45]
PHASE 5	14 (11 upregulated, 3 downregulated) signature genes	Transcriptomics	Prediction of sustained drug-free remission after initiation of methotrexate in DMARD-naive patients with early RA	[45]
PHASE 5	A combination of 3 genes [cytidine monophosphate kinase 2 (CMPK2), IFN-induced protein with tetratricopeptide repeats 1B (IFIT1B), and RNASE3]	Transcriptomics	Prediction of responders and non-responders patients to anti-TNF therapy (ADA, ETP and Golimumab)	[67]
PHASE 5	13- gene expression signature	Transcriptomics	Association with anti-TNF responders (ADA, ETP and Golimumab)	[67]
PHASE 5	10- IFN-regulated genes expression signature	Transcriptomics	Association with anti-TNF nonresponders (ADA, ETP and Golimumab)	[67]
PHASE 5	8-gene expression signature	Transcriptomics	Association with response to anti-TNF therapy	[73]
PHASE 5	Total lymphocyte counts and plasmablast	Non-omics	Association with negative response from Rituximab therapy	[72]
PHASE 5	B cells	Non-omics	Association with positive response to Rituximab	[97]
PHASE 6	MicroRNAs (miRNAs): miR 26b ‐ 5p, miR 487b ‐ 3p y miR 495‐3p	Epigenomics	Association with good response to Allogeneic Adipose‐Derived Mesenchymal Stem Cells therapy	[61]
PHASE 6	MicroRNAs (miRNAs): miR-16-5p, miR-23-3p, miR125b-5p, miR-126-3p, miRN-146a-5p, miR -223-3p	Epigenomics	Significantly associated with positive response to anti-TNFα/DMARDs therapy y parallel to the reduction de TNFα, IL-6, IL-17, RF, CRP	[113]
PHASE 6	GALNT18 C allele SNP (rs4910008)	Genomics	Association with a low disease activity at 6 months in patients previously treated with tocilizumab	[76]
PHASE 6	CD69 A allele SNP (rs11052877)	Genomics	Association with a low disease activity at 6 months in patients previously treated with tocilizumab	[76]
PHASE 6	Matrix metalloproteinase-3 (MMP-3)	Proteomics	Association with radiological progression particularly in early RA. High decrease of this biomarker may indicate better scope of remission; high baseline amounts is associated with clinic response of infliximab	[26, 68–70, 75, 98, 116]
PHASE 6	C–C motif chemokine 22 (CCL22) and C–C motif chemokine 17 (CCL17)	Proteomics	Specific Pharmacodynamics Biomarkers for therapies targeting to Granulocyte Macrophage Colony-Stimulating Factor (GM-CSF)	[36]
PHASE 6	Chemerin	Proteomics	Association with metainflammation and as a clinic modifiable risk factor associated to treatment response	[41]
PHASE 6	Matrix metalloproteinase-8 (MMP-8)	Proteomics	High concentrations in RA chronic patients. MMP-8 levels in saliva was high in eRA patients	[47]
PHASE 6	Matrix metalloproteinase-derived types I, II, and III collagen neoepitopes [C1M, C2M, and C3M]	Proteomics	C1M is associated with radiological progression and both C1M as C3M are associated with treatment efficacy	[48, 69, 109]
PHASE 6	Fibulin-3	Proteomics	Decreased levels during anti-TNF clinic therapy in patients with RA	[49]
PHASE 6	Interleukin 6 receptor (IL6R)	Proteomics	High levels in RA patients are associated with clinical response to tocilizumab	[1, 51, 79]
PHASE 6	Tumor necrosis factor α (TNF-α)	Proteomics	Increased levels in patients with RA. The baseline level is associated with the clinical response to anti-TNF therapy	[1, 51, 53, 55, 71, 79, 116]
PHASE 6	Calprotectin	Proteomics	Association with good or moderate response to RTX and high levels predicts more severe radiological damage after 10 years	[72, 98, 109]
PHASE 6	Intra-Cellular Adhesion Molecule-1 (ICAM-1)	Proteomics	Association with response to anti-TNF therapy	[73]
PHASE 6	Prothrombin fragment F1 + 2and fibrin fragment D-dimer	Proteomics	The reduction of prothrombotic biomarkers parallels the reduction of inflammatory parameters and clinical symptoms in RA patients treated with tocilizumab	[80]
PHASE 6	Interleukin-17 (IL-17)	Proteomics	Significantly higher levels in serum of RA patients. Further, Il-17 is associated to a more active state of disease	[101]
PHASE 6	Soluble gp130 (Spg130), granulocyte macrophage colony-stimulating factor (GM-CSF), interferon gamma-induced protein 10 (IP10)	Proteomics	Prediction of DAS28-CRP score in RA patients not treated with tocilizumab	[102]
PHASE 6	Spg130, Il-6,IP10 and soluble tumor necrosis factor receptor two (sTNFRII)	Proteomics	Prediction of remission in ingenious patients after treatment with tocilizumab	[102]
PHASE 6	Spg130	Proteomics	Prediction of remission in Ra patients treated with tocilizumab at 16 weeks (high levels: > 0,2 μg/ml)	[102]
PHASE 6	Interleukina IL-9 (IL-9), TNF-α, vascular endothelial growth factor (VEGF)	Proteomics	Prediction of DAS28-CRP score at 16 weeks in RA patients treated with etanercept (low fiability)	[102]
PHASE 6	11 metabolites	Metabolomics	Significantly correlated positively or negatively with DAS28-ESR and significantly differed between active and inactive patients	[25]
PHASE 6	Methotrexate polyglutamate	Metabolomics	Measure the response to MTX therapy	[66]
PHASE 6	Concentration parameter calculated as [aspartic acid] + [threonine] + [tryptophan]—[histidine]—[phenylalanine]	Metabolomics	Correlation between painful joints count, inflamed joints count and DAS28 value	[95]
PHASE 6	Dihydrofolate reductase (DHFR), T cell receptor alpha variable 8–3 (TRAV8-3), ephrin receptor A4 (EPHA4) and coiled-coil domain containing 32 (CCDC32)	Transcriptomics	Association with response to tocilizumab therapy (there are expressed after treatment)	[84]
PHASE 6	28 sets of genes (each set contained 22–325 gene probes)	Transcriptomics	Determine the presence of reduced disease activity in response to therapy with anti‐TNF	[111]
PHASE 6	Multi-biomarker disease activity (MBDA)	Non-omics	Calculated based on the 12 different biomarkers (VCAM-1, EGF, VEGF-A, IL-6, TNF-RI, YKL-40, MMP-1, MMP-3, leptin, resistin, SAA, CRP). The score reflects the current clinical activity of the disease	[1, 30, 98]
PHASE 6	Regulatory T cells (Treg)	Non-omics	Monitoring patients but not for predicting their personal response	[62]
PHASE 6	T helper 17 (Th17) cells	Non-omics	Prediction for response to anti-cytokine treatments (Low levels of Th17 cells)	[62]
PHASE 6	Volume transfer constant for Gadolinium-based contrast agent between blood plasma and extravascular (Ktrans/min-1)	Non-omics	Measure the response to biological therapy at 6 weeks	[63]
PHASE 6	Sonography	Non-omics	Measure disease state and predict the relapse and refractary nature of RA	[71]
PHASE 6	Reactive oxygen species and reactive nitrogen species	Non-omics	Effectively serve as biomarkers for monitoring disease progression	[75]
PHASE 6	Residual memory B cells	Non-omics	High levels (Especially memory B cells increased) increase the response risk inadequate or recaid to rituximab therapy	[108]
PHASE 6	Circulating monocytes: CD14+ high CD16− and CD14+ high CD16+ subset cells	Non-omics	Prediction of clinic response reduced to MTX in Ra patients with no treatment previously	[119]

Finally, Table 1 shows biomarkers that can be used as predictors of response to drugs used for treatment of rheumatoid arthritis.

## Discussion

### Current overview of omics-based biomarkers of rheumatoid arthritis in clinical practice

In 2010, new criteria for early classification of RA were published as a joint effort of the European League against Rheumatism/American College of Rheumatology (EULAR) and the American College of Rheumatology (ACR), with the objective to improve the classification in diagnosis based on existing criteria. It was also proposed as a tool to take advantage of therapeutic opportunity in early stages of disease. On the other hand, these new criteria highlight the importance of using biomarkers to support therapeutic decisions in the clinical field [126].

Within these new clinical findings, rheumatoid factor (RF), anti-cyclic citrullinated peptide (ACPA) and acute phase reactants (APR) are so far the most commonly used biomarkers in clinical settings to guide diagnosis and prognosis of RA. And since its description in 1940, RF continues being the most used laboratory tool for diagnosis and prognosis of RA in early stages of disease. However, its use and interpretation has been conditioned since the appearance of ACPAs as more specific markers (95% -98% vs. 85%) and similarly sensitive than RF (60–80% vs. 65%-80%). In addition, the value of ACPA can predict development of disease and response to specific therapies (high levels—low levels).

Despite their wide use, both RF and ACPA, as well as APR (they are nonspecific of the RA and sometimes they do not change with progression) have proved to be insufficient to respond satisfactorily to the high heterogeneity of RA. However, in the last five years omic approaches have had a gradual and homogeneous increase in the discovery and proposal of new biomarkers that could solve difficult questions about decision making in clinical settings.

Consequently, the so far approved biomarker is ideal and robust enough to be the only clinical criteria for diagnosis or prediction of disease with high reliability, specificity, and sensitivity. In this case, not only a biomarker but also a panel or group of biomarkers -that reflect the multifactor nature of the disease state of RA—should be considered. At present, the right choice of candidate biomarkers offers additional and objective information that, when used in conjunction with traditional tools and techniques, represents a potential opportunity to make more informed and integrative clinical decisions that lead to a more precise medical care model. This model will allow stratification of RA patients according to the level of risk and degree of therapeutic opportunity.

In this sense, a patient can benefit from previous diagnosis, stratification in response to severity of disease progress, prediction of response to a specific therapy, and prediction of toxicity reactions or identification of prognostic value avoiding ineffective treatments which can favor exposure to side effects. This allows access to timely care that improves the quality of life and better control of disease, offers a valuable degree of prevention, reduces costs to the health system, and contributes to the possibility of developing new therapies (Table 2).

**Table 2:** Advantage of discovering and using new biomarkers in patients with rheumatoid arthritis

Aim	Function	Advantage
Prevention	Susceptibility	Identification of individuals likely to develop RA
	Protection and exposure of risk	Establishment of mechanisms that can favor maintenance of health status and reduce risk factors
	Risk assessment	Determination of causes, characteristics, traits, possible risks and probable occurrence of unwanted adverse events, as well as their consequences on the onset and development of the disease
Diagnosis	Stratification	Classification of RA patients in different groups to make decisions
	Early diagnosis	Identification of the early state of the disease and contain more successfully its progressive progress
	Better diagnosis	Establishment of the disease with certainty
	Prognostic value	Forecast of disease development and support the therapeutic decision making and clinical benefit from a therapeutic intervention
	Predictive value	Measure patient’s responsiveness to treatment
	Relevance and need	Establishment of time and appropriate measures to address the disease
Treatment	Proper selection	Choice of appropriate drug for specific RA patient
	Response evaluation	Determination of the efficacy of anti-rheumatic therapy
	Disease activity monitoring	Identification of the existence of problematic situations or good evolution of disease and its possible interventions
	Safety	Prevention and/or reduction of side effects that the response to treatment can produce
Value chain	Therapeutic alternatives	Evaluation of recent therapies different from traditional ones
	Development of new drugs	Identification of new therapeutic targets
	Clinical trials optimization	Reduction of costs and time in development of new drugs
	Test development	Improvement of the opportunity and the therapeutic window of medical care

Among the phase 1 biomarkers possess both protective and present actions in patients at risk for RA, this in RF-positive and RF-negative patients. In particular cases such as calgranulin C provides a differentiating characteristic against other inflammatory arthritis (Table 1). As for those proteomics and metabolomics are characterized by their use as clinical diagnostics, and differentiation between RA patients and healthy patients.

The most common associations found in this phase are associated with rapid joint destruction and disease progression. However, there are some omic-biomarkers related with positive outcomes as the requirement of less therapeutic interventions like M1V variant SNP (Table 1). Most of these changes can be assessed by radiological findings.

The major feature that stands out among the phase 6 biomarkers is that they are correlated to treatment response, both for increasing and decreasing biomarker concentration. Their presence can serve as an indicator and predictor of response to treatment with anti-TNF biologic drugs such as ICAM-1. In particular those genomic biomarkers within this phase have an association with reduced disease activity in early RA, for example sTNFRII which is associated with disease remission after treatment with tocilizumab. Among the phase 6 proteomics, relationships are found between a high concentration and clinical response to infliximab such as MMP-3 (Table 1).

### Perspectives and challenges of the use of omics-based biomarkers in clinical practice

Although numerous publications about the discovery of new biomarkers are available, currently, their translation into clinical practice is limited. However, progressive growth of technologies and omic sciences and interest of international organizations such as FDA/EMA, have allowed a better outlook to the use of biomarkers as useful tools to improve quality of healthcare.

Even so, it is necessary to identify and recognize a series of barriers and challenges that must be worked on to have a greater number of biomarkers. In this regard, the following stand out:

- Omics technologies and costs. Although technology is in rapid and progressive growth, they are still expensive.
- Application in clinical practice. It is probably the biggest challenge for clinical application of biomarkers. There is currently a large number of scientific publications on the discovery of new biomarkers, however, the number of biomarkers applied in clinical practice is very low.
- Accessibility, repeatability, and technical validation.
- Validation times. Time from discovery of a biomarker til its validation in clinical practice is usually extensive due to different established requirements.
- Results processing and interpretation. As use of omic technologies grows, it becomes necessary to be able to disseminate and handle a large amount of data that increases in parallel and ensures an added value for patients. Normally, physicians usually focus on a single issue since addressing other technologies would be complicated. In most cases, biomarkers are specific to a population, so it is necessary to establish biomarkers for each group of patients.
- Legal and regulatory matters. Although processes have been initiated to set policies regarding the issue of biomarkers, there are still too many legal gaps to consider.

Process of health care needs parameters to evaluate effectiveness and safety of pharmacotherapy. In this context, biomarkers are an excellent information tool for prevention, diagnosis, identifying progression of disease, selection of treatment and assessment of response to therapy (pharmacodynamics), as well as applications in experimental evaluation [17].

Therefore, it is useful in the application of disease diagnosis, prognostic factor, choice and monitoring of the best possible treatment, and evaluation of therapeutics in a simple, minimally invasive way and without additional risk for the patient [127]. The rapid growth of technological tools, the progression in advances in validation and elucidation of processes and procedures in molecular biology, analytical chemistry and bioinformatics have increased the application of biomarkers in research and later in clinical practice, highlighting omic biomarkers: transcriptomics, genomics, proteomics and metabolomics [128].

Usefulness of using biomarkers for health contributes to selection of medicines, evaluation of progression of diseases and their treatment. In same way, technological developments make it possible for implemented biomarkers to adjust more and more to the concept of an ideal biomarker; that is, they are increasingly specific and fundamental in the development of different biomedical disciplines, Allow the development of strategies and policies that include patients with rheumatoid arthritis and improve their quality of life.

The clinic importance of the biomarkers in RA is still uncertain. This diagnosis is still based on clinical findings and blood tests with non-omic biomarkers. There are many associations that are not totally useful for the diagnosis of RA through omic-biomarkers. Although most of them are related to the characteristics of the disease and their possible outcomes, it has not been possible to perform the diagnostic process with biomarkers alone. However, the usefulness of biomarkers could be established as predictors of disease and outcomes. This could be beneficial in determining the natural history of patients depending on the stage of the disease by personalizing each case.

Therefore, we provide an overview of the pharmacogenomics of RA and the possibility of using omic biomarkers with potential to be used in clinical practice and to support pharmacotherapeutic decisions in order to improve response and safety to treatment.

The relevance of this study lies in providing the possibility to encourage the investigation of omic biomarkers -selected for their biological importance in AR- either in pharmacokinetic and pharmacodynamic processes, to provide additional tools that facilitate the identification of individuals at risk of suffering adverse events or individuals likely to fail treatment. Therefore, it is expected that the information generated can be used in daily clinical practice, helping to choose the best therapeutic option, at the right time with the least possible risk (greater effectiveness and safety) in patients with rheumatoid arthritis [8].

## Conclusions

Globally, there is not a totally effective medication in all patients, and each individual has a different response to drug treatment. This could be explained due to a modification in pharmacokinetics and pharmacodynamics properties of drugs related with genetic environmental conditions. In this context, the investigation of omic biomarkers has been more successful in the identification and explanation of the alteration of pharmacological response, compared to investigations of candidate genes of disease. Therefore, this paper should make a contribution to the selection of the best therapeutic management in patients with RA according to the phase of disease and is a basis to continue the research aimed at the identification of omic biomarkers according to stage of RA and treatment phase.

As observed in this systematic review, in the last decade a great effort has been made to find omic biomarkers capable of predicting the response to therapy in a patient with rheumatoid arthritis. Many biomarkers have been explored and, even though several omic biomarkers have been identified, there are limitations with respect to their specificity, ease of sampling, representativeness, and stability to predict response. Thus, more comprehensive research is still needed in the identification of omic biomarkers in different phases of rheumatoid arthritis with promising next-generation sequencing and nuclear magnetic resonance techniques.
